# Psychological health problems during the lockdown: A survey of Indian population in COVID-19 pandemic

**DOI:** 10.1016/j.dib.2020.106566

**Published:** 2020-11-23

**Authors:** Blessy Elizabeth David, Sanjay Kumar

**Affiliations:** a Doctor Harisingh Gour Vishwavidyalaya, Sagar, Madhya Pradesh, India; b Doctor Harisingh Gour Vishwavidyalaya, Sagar, Madhya Pradesh, India

**Keywords:** Mental health, Coronavirus, Depression, Anxiety, Social dysfunction, Lockdown, Pandemic

## Abstract

The global crisis prevailing in the wake of the spread of COVID-19 has raised several speculations about the impact of the lockdown on the mental health of people. The dataset presented here is the assessment of the psychological distress experienced by people in India following the implementation of lockdown as a measure to curtail the spread of the coronavirus. The data was collected through a survey conducted by employing an online questionnaire assessing the socio-demographic information (9-items) as well as the administration of the short version of the General Health Questionnaire (GHQ-12 items) originally developed by Goldberg (1972). The period of data collection is between 9th April 2020 and 20th April 2020 where a total of 1,894 responses were obtained. The Google forms containing the questionnaire of the study were shared publicly through emails and via the social media forum like WhatsApp and Facebook. Thereby, those who took the initiative to fill-up the responses were included as the survey participants. Thus, the final sample had participants representing 17 states and Union territories of India. The entire dataset is stored in a Microsoft Excel Worksheet (.xls) and the questionnaire is attached as a supplementary file. The data is beneficial for the timely assessment of the nature and degree of the psychological distress experienced by people in India during the COVID-19 crisis. It could further be an assistance to the Government, policymakers as well as health care workers to take the adequate measures to ensure sound mental health among people.

## Specifications Table

**Table:** 

Subject	Public Health
Specific Subject area	Health Psychology, Clinical Psychology, Social Psychology
Type of data	Primary data and Tables
How the data was acquired	The data was obtained employing online questionnaires (Google forms) circulated publicly through emails and via the social media platforms of WhatsApp and Facebook. The questionnaire is included in this article and maybe accessed online via the following link- https://forms.gle/pm528gyxBwGLrRus9
Data format	Raw, Analyzed, Filtered (descriptive statistics)
Parameters for data collection	The survey data were obtained from respondents in 17 Indian states and Union territories who had access to the internet connection as well as social media.
Description of data collection	The link to the online questionnaire (Google forms) was shared publicly through email and the social media platforms of WhatsApp and Facebook. Those who came across the forms and took the time to respond to it were included as research participants. The questionnaire was circulated in bilingual mode with English and Hindi for the ease of the respondents from different geographical locations of India.
Data source location	The research participants represent 17 different states and Union territories of India.
Data accessibility	The dataset is uploaded on Open Science Framework Repository Name: Open Science Framework Direct URL to the data https://osf.io/gey46/?view_only=6dc98f804b424a46bed824f02c60ea5f

## Value of the Data

• The data represents a pool for the exploration of COVID-19 related information, family interaction related information, and information regarding the general health of the adolescents and young adults from the Indian sub-continent.
• The present data can be useful to compare with other similar studies on family interaction and information regarding general health from other countries across the globe. The dataset would contribute to serving as a heuristic basis for further insight into the phenomena of the COVID-19.
• Researchers can utilize the data in the future by employing statistical analysis to examine the relationships between Sociodemographic factors, COVID-19 related knowledge, family interaction related information, and general health information during the present crisis.
• The findings of the analyzed data may be beneficial for preventing and curbing the spread of COVID-19. Besides, the data can also assist in the planning for public health interventions as well as policy formulation and implementation. This dataset would also be beneficial for the psycho-education of the common masses who are living under extreme stress conditions today.

## 1 Data Description

The dataset is an assessment of the psychological distress reported by the respondents from 17 different states and Union territories of India during the lockdowns implemented to contain the COVID-19. The obtained raw data for the information presented in each table is secured in a Microsoft Excel Worksheet (.xls). Items 1–8 contain information about the respondents’ age, gender, level of education, type of residence, religion, relationship status, family's income status, number of members in the family descriptively presented in Table 1. In the table, the respondents’ age has been categorized into different age-groups. The education level has been demarcated into groups mentioning the highest level achieved by the respondents based on the schooling levels practiced in India. The place of residence has been grouped as urban- describing the respondents hailing from the developed sectors of the society. On the other hand, the rural category pertains to the ones hailing from the under-developed sections of the society. Religion has been classified based on the responses of the participants. The ‘others category’ stands for those choosing not to disclose their religious identity or identifying with any other indigenous religion in India. Relationship status refers to the present state of romantic affiliations in the respondents’ life. Since the family structure in India is collectivistic predominantly, therefore the Family's annual income refers to the income generated within the respondents’ family annually combining all the resources measured in Indian rupees (INR). Similarly, the category mentioning the number of family members is the reference to the members in the immediate family of the respondents inclusive of parents and siblings. Table 2 represents the time frame when the respondents became aware of the present pandemic. The table categorizes the respondents based on their awareness levels as they responded to the question in item-9 as “When did you first come to know about the pandemic?”

**Table 1:** Descriptive statistics of sample characteristics (*n* = 1894).

Variables	Categories	Frequency	Percentage
Age	Below 18 years	163	08.60
	18–25	1329	70.20
	26–33	259	13.70
	34–39	42	02.20
	40–46	41	02.20
	47–53	36	01.90
	54–60	12	0.60
	61–69	7	0.40
	70–75	1	0.10
	76–80	2	0.10
	Above 80	2	0.10
Gender	Male	1119	59.10
	Female	775	40.90
Level of Education	Middle School	989	52.20
	Secondary School	419	22.10
	Senior Secondary	26	1.40
	Graduation	281	14.80
	Post-graduation	112	5.90
	Doctoral level	43	2.30
	Post-doctoral level	24	1.30
Type of residence	Rural	756	39.90
	Urban	1138	60.10
Religion	Hinduism	1626	85.90
	Islam	42	2.20
	Christianity	23	1.20
	Jain	174	9.20
	Others	29	1.50
Relationship Status	Single	1533	80.90
	Married	272	14.40
	Committed/ dating	84	4.40
	Divorced	4	0.20
	Widow/ widower	1	0.10
Family annual income status (in rupees)	00,000–12,000	265	14.0
	12,000–36,000	401	21.2
	37,000–100,000	401	21.2
	100,001–200,000	170	9.0
	200,000–300,000	189	10.0
	300,001–400,000	121	6.40
	400,001–500,000	118	6.20
	500,001–600,000	57	3.00
	600,001–700,000	42	2.20
	700,001–800,000	30	1.60
	800,001–900,000	15	0.80
	900,001–1,000,000	24	1.30
	1,000,001 and Above	61	3.20
Number of members in the family	2 members	57	3.00
	3 members	196	10.30
	4 members	648	34.20
	5 members	516	27.20
	more than 5 members	477	25.20

**Table 2:** Descriptive statistics representing the timeline when the respondents received information about COVID-19 (*n* = 1894).

Awareness about COVID-19 (Month)	Frequency	Percentage
December	264	13.9
January	449	23.7
February	416	22.0
March	739	39.0
April	27	1.5

The detailed assessment of the psychological discomforts experienced by the participants is represented in Table 3. Items 10–21 were from the General Health Questionnaire (GHQ-12 items) originally developed by Goldberg [1]. The respondents were asked to reflect the time since the implementation of lockdown in India (late March 2020) and indicate their responses to each item of the GHQ. The scoring pattern has been followed with respect to the codes 0–1–2–3 for each response of Always, Sometimes, Occasionally and Never based on whether the item is positively worded or negatively worded [2]. The responses to each item have been described in detail in the given table.

**Table 3:** Descriptive statistics on the responses to the items of the General Health Questionnaire (GHQ-12 items) in percentage (*n* = 1894).

Items	Responses				Statistics	
Since the implementation of lockdown, I experienced that	Always	Sometimes	Occasionally	Never	Mean	S.D.
I am able to concentrate on things	61.9	30.0	5.4	2.6	0.49	0.717
I am losing my sleep due to worry	7.6	35.5	13.1	43.8	1.07	1.045
Playing a useful part in everyday life	54.6	30.9	7.8	6.7	0.67	0.882
I am capable of making decisions	70.7	22.5	4.3	2.5	0.39	0.688
I constantly funder strain	6.1	33.1	21.6	39.2	1.06	0.982
I am unable to overcome my difficulties	14.6	33.2	14.8	37.4	1.25	1.108
I am unable to enjoy day-to-day activities	56.9	28.3	8.2	6.6	0.65	0.889
I am able to face problems	69.4	22.3	5.0	3.2	0.42	0.733
I am feeling unhappy and depressed	5.4	39.1	19.2	36.2	1.14	0.976
I am losing my confidence	3.3	25.1	13.0	58.6	2.27	0.948
I am thinking of myself as worthless	4.1	19.3	9.7	67.0	0.60	0.932
I am feeling reasonably happy	38.3	39.5	11.4	10.8	0.95	0.962

## 2 Survey Design, Materials and Methods

The research employed the descriptive survey design to study the influence of lockdown during the pandemic on the mental health and psychological functioning of the respondents. The dataset includes 1894 responses collected between 9th April 2020 and 20th April 2020 following the implementation of the nationwide lockdown as a measure to contain the spread of COVID-19. This study was approved by the departmental Human Ethics Review Board of Doctor Harisingh Gour Central University, Sagar, India. The respondents’ participation was completely consensual, anonymous, and voluntary. The Google form was designed such as to keep the personal details of the responded confidential if he/she wishes to, however it was open for those who wished to reveal their personal details in the online form. While, the forms were shared publicly to the respondent, they were given the flexibility to refuse from participating in the present study. No force was used compelling them to submit the responses in the online questionnaire.

Due to the physical constraints of the implementation of lockdown to contain the spread of COVID-19 in India, we relied upon the online mode of data collection for the present study. Studies have previously supported the online mode of data collection for situations where the space and time constraints do not facilitate the face-to-face interaction for the research purpose [3]. Google forms were used in the present study to avail the advantages of unlimited surveys and respondents within a single study. Another added feature is the automated display of the collected data in the form of Microsoft excel spreadsheet for clarity and conciseness. Google forms being a widely used and familiar mode of survey in India, were employed in the present study accounting for the user comfort and familiarity of the respondents [4]. An online questionnaire (Google form) was prepared and circulated in public through the emails and the social media platforms (WhatsApp and Facebook) to seek responses from the participants (See Supplementary material). The first eight items of the form primarily seek the demographic information about the participants namely- the respondents’ age, gender, level of education, type of residence, religion, relationship status, family's income status, and the number of members in the family. Item-9 attempts to assess the awareness of the participants by eliciting the response as to when they came to know about COVID-19 for the first time. Items 10–21 (Section B) pertain to the General Health Questionnaire (Short version-12 items) initially prepared by Goldberg (1972) and adapted for the Indian population by [5]. The Cronbach Alpha for the short version of the GHQ (12-items) is 0.844. The items require the participants to express their psychological functioning since the implementation of the lockdown in India. The 12-item scale contained both positively worded items (Items- 1, 3, 4, 7, 8, 10, 12) which were scored on a scale of 0–3 as Always- 0, Sometimes- 1, Occasionally- 2 and Never- 3. Similarly, the negatively worded items (Items- 2, 5, 6, 9, 11) were scored on a reverse scale of 3–0 as Always- 3, Sometimes- 2, Occasionally- 1 and Never- 0 [2]. On the whole, the higher total scores on the GHQ indicate higher psychological discomfort experienced by the respondents and vice-versa.

The dataset incorporating the demographic information as well as the participants’ responses to the perceived psychological discomforts employing the GHQ during the lockdown was analyzed using the frequency distribution table as well as by computing the percentage, means, and standard deviation for the elicited responses on the Google form.

## Declaration of Competing Interest

The present research did not receive financial support in any form from any institutions/ authorities for the conduct/ publication of the research work. The authors declared no potential conflicts of interest concerning the research, authorship, and/or publication of this article.
